# Icaritin Provokes Serum Thrombopoietin and Downregulates Thrombopoietin/MPL of the Bone Marrow in a Mouse Model of Immune Thrombocytopenia

**DOI:** 10.1155/2018/7235639

**Published:** 2018-08-27

**Authors:** Ke Zhang, Zhenfeng Dai, Runzhe Liu, Fang Tian, Xi Liu, Yi Sun, Xin Zhao, Xiaoping Pu

**Affiliations:** ^1^National Key Research Laboratory of Natural and Biomimetic Drugs, Peking University, Beijing 100191, China; ^2^Department of Molecular and Cellular Pharmacology, School of Pharmaceutical Sciences, Peking University, Beijing 100191, China

## Abstract

Immune thrombocytopenia (ITP) is a common acquired autoimmune disease, and thrombopoietin (TPO) is an important cytokine that regulates the production of megakaryocytes and platelets. We have identified a biologically active component, icaritin, from a Chinese herba epimedii extract. Icaritin promotes platelet production and regulates T cell polarization, but its mechanism is not clear. In this study, the BALB/c mouse model of ITP was established by injection of an antiplatelet antibody every other day for seven total times. The antiplatelet sera were derived from guinea pigs immunized with the platelets of BALB/c mice. Mice with ITP were treated with icaritin at low, moderate, or high doses of 4.73, 9.45, and 18.90 mg/kg, respectively, for fourteen consecutive days. The present study shows that icaritin can significantly increase peripheral blood platelet counts and thrombocytocrit, increase the TPO level in serum, attenuate splenomegaly, and reduce the abnormal proliferation of megakaryocytes in the spleen and bone marrow. Icaritin can also downregulate the expression of bone marrow TPO, myeloproliferative leukemia virus oncogene (MPL), and p-Stat3. Our results suggest that icaritin can significantly improve the health of mice with ITP via possible downregulation of p-Stat3 expression in the JAK2/Stat3 phosphorylation signaling pathway and regulation of bone marrow TPO/MPL metabolism.

## 1. Introduction

Immune thrombocytopenia (ITP) is also known as autoimmune thrombocytopenic purpura or idiopathic thrombocytopenic purpura, and it is a common acquired autoimmune bleeding disorder. Decreased peripheral platelet counts and bleeding are its primary clinical manifestations [1]. Autoantibody-mediated humoral immunity-induced platelet destruction is important in the pathogenesis of this disease [2]. ITP accounts for approximately 1/3 of the bleeding disorders. The incidence of ITP is approximately 2 to 4 cases per 100,000 adults [3]. Elderly people over the age of 60 years are the high-incidence population for this disease, which causes extensive skin and mucous membrane bleeding and epistaxis. Severe cases cause visceral and intracranial hemorrhages, which endanger life [4].

Thrombopoietin (TPO) is the key cytokine that promotes the production of megakaryocytes and platelets, and it is primarily synthesized in the liver [5]. The TPO receptor in vivo is the myeloproliferative leukemia virus oncogene (MPL), also known as CD110. The expression of MPL plays an important role in the regulation of megakaryocyte production. MPL overexpression produces excessive megakaryocyte proliferation and increased platelet production. Megakaryocyte proliferation is attenuated when MPL expression is insufficient or absent, which leads to the development of thrombocytopenia [6]. Serum TPO levels are used to differentiate the etiology of thrombocytopenia and evaluate the effect of TPO agonist treatment [7]. TPO in combination with MPL activates a series of signaling pathways, such as JAK/Stat, PI3K/Akt, and Ras/MAPK [8], which transmit extracellular signals to the nucleus and promote the replication, maturation, and differentiation of megakaryocyte progenitor cells, thus increasing the number of platelets in peripheral blood [9–11].

**Figure 1:**
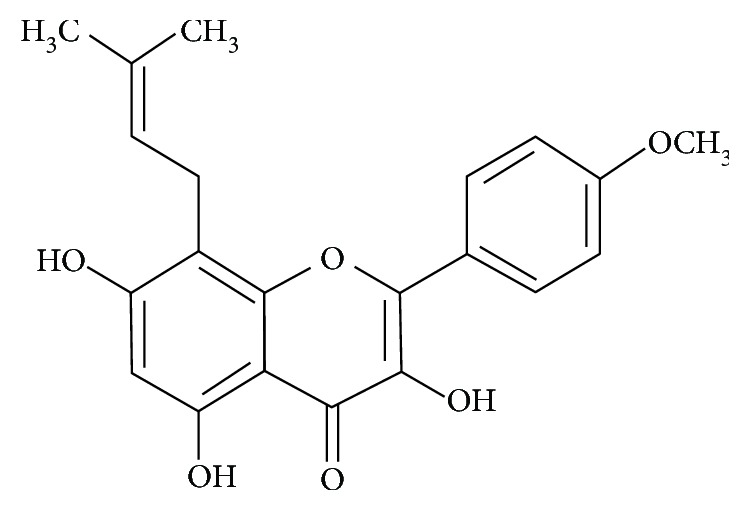
Chemical structure of icaritin.

Icaritin is a monomeric compound that is produced by the hydrolysis of icariin, a flavonoid component extracted from *Epimedium* Linn.  Figure 1 shows the chemical structure of icaritin. Icaritin promotes hematopoietic function, inhibits tumor cell differentiation, improves immune function, regulates the endocrine system, and exhibits inflammatory activity [12–16]. Nanooral preparations of icaritin were created to improve its absorption rate, and these preparations promote platelet production and regulate T cell polarization [15]. The present study investigated the effect of icaritin nanopreparations (Y003) in mice with ITP and elucidated its mechanism on megakaryocytes, TPO, and MPL in the spleen and bone marrow.

## 2. Materials and Methods

### 2.1. Animals

Male BALB/c mice (SCXK (Jing) 2016-0011, Beijing, China) (6–8 weeks old, weighing 19 to 24 g) and male Hartley guinea pigs (SCXK (Jing) 2016–0001, Beijing, China) (6 weeks old, weighing 300–400 g) were purchased from Beijing Vital River Laboratory Animal Technology Co. Ltd. Mice were maintained at a controlled temperature (24 ± 2°C) and housed in separate cages (12 h light/dark cycle) with access to food and water ad libitum. The animal ethics committee of Peking University approved all the experimental procedures. The Animal Care and Use Committee of Peking University approved all the experimental procedures involving the use of animals.

### 2.2. Preparation of Mice with ITP

Guinea pig anti-mouse platelet antiserum (GP-APS) was prepared according to reported methods [17, 18] with some modifications. Animals were anesthetized, and blood was collected in plastic tubes containing EDTA-K_2_ (15 mg/mL; AMRESCO, USA) and centrifuged at 1000 r/minute for 10 minutes, and the supernatant (platelet-rich plasma) was centrifuged at 3000 r/minute for 10 minutes. The supernatant was discarded, and the platelets were precipitated. The precipitate was washed three times with PBS, and the supernatant was discarded. A platelet suspension of 10^9^ cells/mL was prepared using physiological saline. The platelet suspension was mixed with an equal volume of complete Freund's adjuvant or incomplete Freund's adjuvant (Sigma, USA) via ultrasound. Blood with EDTA-K_2_ was centrifuged at 800 r/minutes for 10 minutes, and the precipitate containing erythrocytes was collected. Saline was added to prepare a 5% erythrocyte suspension. The platelet suspension containing complete Freund's adjuvant was injected subcutaneously to at least 4 points in the paw and to the back and abdomen of guinea pigs in the first week. The platelet suspension containing incomplete Freund's adjuvant was injected in the same manner in the subsequent 4 weeks. Each injection volume was 1 mL/body. The guinea pig sera containing antiplatelet antibodies were collected in the sixth week. Guinea pig sera were heated at 56°C for 30 minutes to inactivate the complement. An equal amount of 5% BALB/c mouse erythrocyte suspension was added, and the mixture was incubated at 37°C for 1 hour. The mixture was centrifuged at 3000 r/minute for 10 minutes, and the precipitate was discarded. This step was performed twice to prepare a 1 : 4 GP-APS. The 1 : 4 GP-APS was stored at −20°C prior to use.

### 2.3. Animal Grouping and Treatment

Sixty BALB/c mice were randomly divided into 6 groups according to body weight, with 10 mice per group. The 1 : 4 GP-APS at room temperature was injected intraperitoneally into BALB/c mice (100 *μ*L per mice) every other day in all groups except for the blank control mice for seven total times. After GP-APS was given an hour later, we administered Y003 intragastrically each day for 14 consecutive days at low, moderate, and high dosages of 4.73, 9.45, and 18.90 mg/kg, respectively. The rhTPO group was subcutaneously administered at 30000 U/kg daily. The control and model groups were administered with saline solution.

### 2.4. Blood Cell Count

Blood was collected from the orbital vein of the mice at the end of the experiment and mixed with anticoagulant dilution. Blood cell count was measured using a MEK-6318K blood cell analyzer (Nihon Kohden, Japan) within 2 hours, and parameters in the peripheral blood of each group were analyzed.

### 2.5. Determination of TPO in Serum Using the Enzyme-Linked Immunosorbent Assay

Serum concentration of TPO was measured by an enzyme-linked immunosorbent assay (ELISA, R&D System, USA) according to the instructions of the manufacturer. Differences in serum TPO levels between groups were analyzed.

### 2.6. Spleen Index

Mice were weighed and sacrificed at the end of the experiment. The spleens were immediately collected and weighed. Spleen appearance and morphology were checked for congestion. The spleen index was calculated using the following formula: spleen index = spleen wet weight/body weight. Differences between groups were analyzed.

### 2.7. Histopathological Examination of the Spleen and Femur

The spleen and left femur were isolated at the end of the experiment and stored in a 4% paraformaldehyde solution for 48 hours. Samples were subjected to routine dehydration and sliced into 5 *μ*m paraffin sections. Hematoxylin-eosin staining (Servicebio, Wuhan, China) was performed, and the morphological structures of the spleen, femur, and megakaryocytes were observed under an optical microscope according to reported methods [17, 18] with some modifications. Six random fields were observed in each slice under a 200x microscope for megakaryocyte counts. The numbers of megakaryocytes in the femur and spleen were analyzed.

### 2.8. Detection of TPO Expression Using Immunohistochemistry of the Femur [19]

Paraffin sections of the femur were routinely dewaxed, rehydrated, and placed in EDTA antigen retrieval buffer (pH 8.0; Servicebio, Wuhan, China). Sections were cooled, rinsed with PBS, and incubated in 3% aqueous hydrogen peroxide (Servicebio, Wuhan, China) at room temperature in the dark for 25 minutes to block endogenous peroxidase activity. Sections were rinsed with PBS and blocked with 3% BSA (Servicebio, Wuhan, China) at room temperature for 30 minutes. Sections were incubated with a primary anti-TPO rabbit polyclonal antibody (1 : 200, Abcam, UK) overnight at 4°C. After primary antibody incubation, sections were rinsed with PBS, incubated with a peroxidase-conjugated goat anti-rabbit secondary antibody (1 : 200, KPL, USA) for 50 minutes at room temperature, visualized with diaminobenzidine, and counterstained with hematoxylin. Three random fields per slice were observed under a 200x microscope, and TPO expression was analyzed.

### 2.9. Detection of MPL Expression Using Immunohistochemistry of the Femur

Samples were pretreated in the same manner described in Section 2.8. An anti-MPL rabbit polyclonal antibody (1 : 200, Bioss, China) was used as the primary antibody, and the sections were incubated at 4°C overnight. Sections were rinsed with PBS, incubated with a peroxidase-conjugated goat anti-rabbit secondary antibody (1 : 200, KPL, USA) for 50 minutes at room temperature, visualized with diaminobenzidine, and counterstained with hematoxylin. Three random fields in each slice were observed under a 200x microscope, and the number of MPL-positive megakaryocytes was analyzed according to previously reported methods with some modifications [20].

### 2.10. Expression of p-Stat3 Using Immunohistochemistry of the Femur

Pretreatment was performed as described in Section 2.8. Sections were incubated overnight with the primary antibody p-Stat3 rabbit polyclonal antibody (1 : 200, Abcam, UK) at 4°C. After primary antibody incubation, the sections were rinsed with PBS, incubated with a peroxidase-conjugated goat anti-rabbit secondary antibody (1 : 200, KPL, USA) for 50 minutes at room temperature, visualized with diaminobenzidine, and counterstained with hematoxylin. Three random fields per slice were observed under a 200x microscope, and the number of p-Stat3-positive megakaryocytes was statistically analyzed according to previously methods with some modifications [18].

### 2.11. Statistical Analysis

Peripheral blood cell counts are expressed as the mean ± SD, and other results are expressed as mean ± SEM. The significance of the difference between groups was analyzed using one-way ANOVA.

## 3. Results

### 3.1. Blood Cell Count Results

Table 1 shows the results of peripheral blood cell counts. PLT and PCT in the peripheral blood were significantly decreased in the model group compared to the control group (both *P* values < 0.001). PLT and PCT were significantly increased in the rhTPO-treated group compared to the model group (*P* < 0.001, *P* < 0.05). PLT and PCT in the low, middle, and high doses of icaritin groups were also significantly increased (three *P* values < 0.001). These results demonstrate that icariin significantly increased platelet counts in the peripheral blood of ITP mice and improved the symptoms of thrombocytopenia. Continuous injection of GP-APS resulted in greater platelet destruction in peripheral blood and increased MPV (*P* < 0.001), and low doses of icaritin reduced MPV (*P* < 0.05). MCV and MID increased significantly (*P* < 0.001, *P* < 0.01), and MCHC decreased significantly (*P* < 0.01) in the model group compared with the control group. The MCV, MID, or MCHC of ITP mice have no changes with the icaritin administration.

### 3.2. Testing Serum TPO Levels Using ELISA

**Figure 2:**
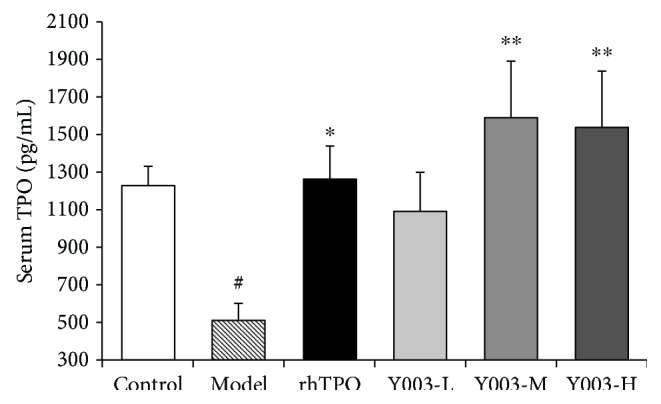
Icaritin promoted serum TPO expression in ITP mice. Data are presented as mean ± SEM (*n* = 6 per group). One-way ANOVA was used to analyze differences among the groups. ^#^*P* < 0.05 versus control group; ^∗^*P* < 0.05 and ^∗∗^*P* < 0.01 versus model group.

Figure 2 shows the results of serum TPO levels. TPO levels in the model group decreased compared to those in the control group (*P* < 0.05). TPO levels in the rhTPO-treated group increased compared to those in the model group (*P* < 0.05). TPO levels were significantly increased (*P* < 0.01, *P* < 0.01) in middle and high doses of the icaritin-treated groups compared to the model group. These results show that icaritin promoted serum TPO expression in ITP mice.

### 3.3. Spleen Index

**Figure 3:**
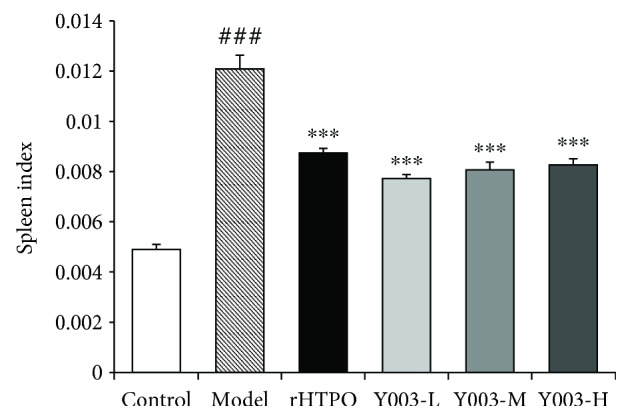
Icaritin alleviated splenomegaly in ITP mice. Data are presented as mean ± SEM (*n* = 10 per group). One-way ANOVA was used to analyze differences among the groups. ^###^*P* < 0.001 versus control group; ^∗∗∗^*P* < 0.001 versus model group.

Figure 3 shows the results of the spleen index. The spleen index in the model group was significantly hypertrophied, and the spleen index increased significantly compared to that in the control group (*P* < 0.001). The spleen index was significantly lower in the rhTPO-treated group than in the model group (*P* < 0.001). The low, middle, and high doses of icaritin-treated groups revealed significantly lower spleen indexes (three *P* values < 0.001). These results demonstrate that icaritin significantly improved the symptoms of spleen hypertrophy in ITP mice.

### 3.4. Splenic Histopathology

**Figure 4:**
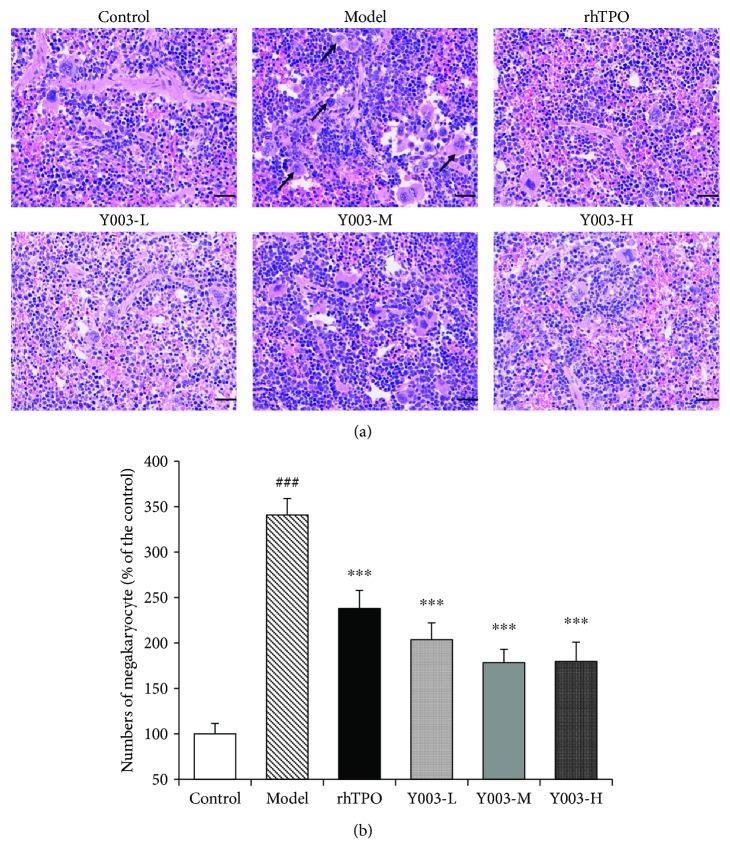
Icaritin improves the abnormal proliferation of megakaryocytes in the spleen of ITP mice. (a) HE staining of the spleen. Black arrows indicate megakaryocytes. (b) Icaritin decreased the number of megakaryocytes in the spleen of ITP mice. Data are presented as mean ± SEM (*n* = 5 per group). Scale bar = 50 *μ*m, ×200. One-way ANOVA was used to analyze differences among the groups. ^###^*P* < 0.001 versus control group; ^∗∗∗^*P* < 0.01 versus model group.

Figure 4(a) shows the HE staining results of the spleen. The number of megakaryocytes in the spleen increased significantly in the model group compared to the control group (*P* < 0.001). The number of megakaryocytes in the spleen was significantly lower in the rhTPO-treated group than in the model group (*P* < 0.001). The numbers of megakaryocytes in the spleen were also significantly reduced (three *P* values < 0.001) in the three icaritin-treated groups compared to the model group (Figure 4(b)). Histopathology demonstrated that icaritin significantly reduced the number of spleen megakaryocytes in mice with ITP and improved the proliferation of splenic megakaryocytes.

### 3.5. Femoral Histopathology

**Figure 5:**
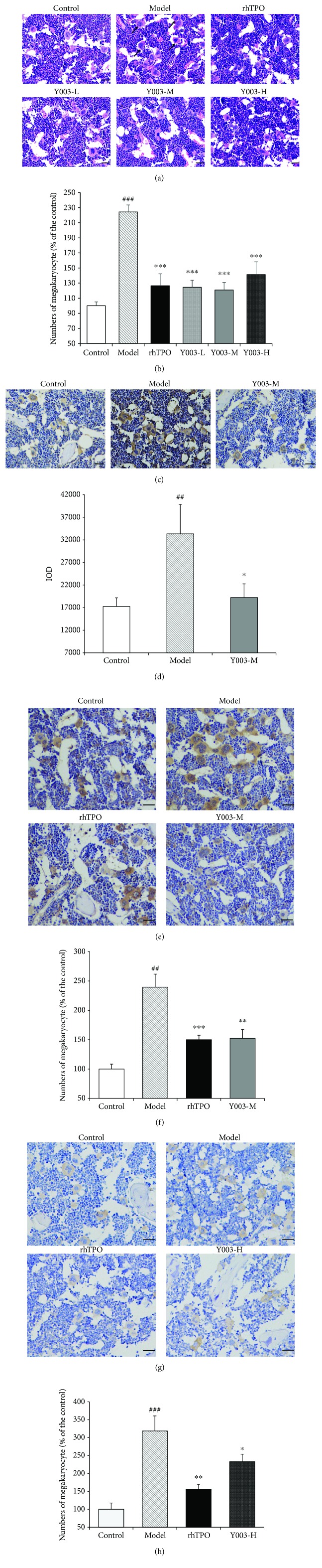
Icaritin is the active ingredient that improves the abnormal proliferation of megakaryocytes and invokes the TPO/MPL axis in the bone marrow of ITP mice. (a) HE staining of the bone marrow. Black arrows indicate megakaryocytes. (b) Icaritin decreased the number of megakaryocytes in the bone marrow of ITP mice. (c) TPO immunohistochemical staining in the bone marrow. (d) Icaritin inhibited TPO protein expression. (e) MPL immunohistochemical staining in the bone marrow. (f) Icaritin decreased MPL protein expression in megakaryocytes. (g) p-Stat3 immunohistochemical staining in the bone marrow. (h) Icaritin inhibited p-Stat3 protein expression in megakaryocytes. Data are presented as the mean ± SEM (*n* = 3–5 per group). Scale bar = 50 *μ*m, ×200. One-way ANOVA was used to analyze the difference among the groups. ^##^*P* < 0.01 and ^###^*P* < 0.001 versus control group; ^∗^*P* < 0.05, ^∗∗^*P* < 0.01, and ^∗∗∗^*P* < 0.01 versus model group.

Figure 5(a) shows the HE staining results of the femur. The number of megakaryocytes in the bone marrow increased significantly in the model group compared to the control group (*P* < 0.001). The number of bone marrow megakaryocytes was significantly lower in the rhTPO-treated group than in the model group (*P* < 0.001). The low, middle, and high doses of icaritin-treated groups revealed a significant reduction (three *P* values < 0.001) in the number of bone marrow megakaryocytes compared to the model group (Figure 5(b)).  Figure 5(c) shows the femoral TPO immunohistochemistry. TPO expression in the bone marrow increased significantly in the model group compared to the control group (*P* < 0.01). TPO expression in the bone marrow was lower in the medium dose of the icaritin-treated group than in the model group (*P* < 0.05) (Figure 5(d)).  Figure 5(e) shows the femur MPL immunohistochemistry results. The number of MPL-positive megakaryocytes in the bone marrow increased significantly in the model group compared to the control group (*P* < 0.001). The number of MPL-positive megakaryocytes decreased significantly in the bone marrow of the rhTPO-treated group compared to the model group (*P* < 0.001). The number of MPL-positive megakaryocytes in the bone marrow was significantly lower in the middle dose of the icaritin-treated group than in the model group (*P* < 0.01) (Figure 5(f)).  Figure 5(g) shows the results of p-Stat3 immunohistochemistry in the bone marrow. The number of p-Stat3-positive megakaryocytes in the bone marrow increased significantly in the model group compared to the control group (*P* < 0.001). The number of p-Stat3-positive megakaryocytes increased significantly in the bone marrow of the rhTPO-treated group compared to the model group (*P* < 0.01). The number of p-Stat3-positive megakaryocytes in the bone marrow was lower in the icaritin high-dose group than in the model group (*P* < 0.05) (Figure 5(h)). These results demonstrate that icaritin significantly reduces the number of bone marrow megakaryocytes, improves bone marrow megakaryocyte hyperplasia, downregulates the expression of TPO and MPL protein, and inhibits the expression of p-Stat3 in the JAK2/Stat3 signaling pathway in ITP mice.

## 4. Discussion

Platelets are differentiated from megakaryocytes. One megakaryocyte can produce thousands of platelets [21]. Most ITP patients exhibit bone marrow megakaryocyte hyperplasia accompanied by abnormal megakaryocyte differentiation and maturation. The spleen is the body's largest immune organ and produces anti-platelet autoantibodies and destroys and stores platelets. The number, morphology, and maturity of bone marrow and spleen megakaryocytes are important indicators in determining disease efficacy [22].

Injection of antiplatelet serum (APS) is a common passive immunization modeling method. The continuous administration of exogenous antiplatelet serum to stimulate the immune system to destroy peripheral platelets reduces the platelet count. This modeling method simulates the pathogenesis and clinical manifestation of human ITP autoimmunity, and it is a simple model that exhibits good repeatability and low cost; this model is widely used to evaluate the efficacy of ITP [23]. The present study used a chronic sustained ITP model in mice using guinea pig anti-mouse platelet serum. Peripheral blood platelet parameters are important indicators in the diagnosis and treatment of ITP [22]. The present study used a continuous injection of GP-APS, which resulted in greater platelet destruction in the peripheral blood. PLT and PCT of mouse peripheral blood decreased significantly with respect to the control group. This difference was statistically significant, but there was no significant difference in the number of red blood cells, white blood cells, or hemoglobin between groups, which is consistent with the pathological appearance of human ITP blood and indicates that the model was successfully prepared [22]. The number of platelets in the icaritin-treated group and the rhTPO-treated group increased significantly, which indicates that icaritin improved ITP.

Neonatal platelets are primarily stored in the spleen and regulate the amount of platelets in peripheral blood. Antiserum injection resulted in spleen hyperplasia in the present study, and treatment significantly reduced the degree of spleen hypertrophy and protected platelets. HE staining-detected changes are megakaryocyte and parallel megakaryocyte numbers in the spleen and femur [24, 25]. The results demonstrated that megakaryocytes in the spleen and bone marrow of the model group increased significantly compared to those of the control group, and a reduction in the production of plate-type megakaryocytes was observed, which indicates that ITP mice exhibited megakaryocyte proliferation and maturation disorders, which is consistent with the pathological characteristics of human ITP [26]. These observations may be due to decreased platelet levels in peripheral blood, which resulted in a compensatory proliferation of megakaryocytes in vivo. However, the presence of antiplatelet antibodies affects the maturation and differentiation of megakaryocytes. Icaritin treatment significantly reduced megakaryocytes compared to the model group. Megakaryocytes increased with respect to the control group, which indicates that icaritin regulated the production of megakaryocytes, improved bone marrow and spleen megakaryocytic hyperplasia, and promoted the recovery of the hematopoietic system.

TPO is a very important regulatory factor for the megakaryocyte proliferation, differentiation, and maturation and platelet production. As a TPO receptor agonist, rhTPO was considered a positive drug in this study, in order to evaluate whether rhTPO and icaritin had similar effects in the treatment of ITP. The combination of TPO and its unique specific functional receptor MPL promoted the proliferation and differentiation of megakaryocytes and hematopoietic stem cells and maintained hematopoietic balance in the body. The results of the present study show that serum TPO levels decreased in the model group and TPO content significantly increased in the icaritin-treated group. The interaction of TPO/MPL affects megakaryocyte proliferation and differentiation and the expression of TPO in serum [27]. TPO and MPL form a complex that is internalized in megakaryocytes, and the proteasome degrades the platelets. Therefore, platelet levels decreased with the decrease in serum TPO levels in ITP mice. The modeling method in the present study destroyed the platelet counts in the peripheral blood, reduced the platelet count, increased the hematopoietic function of the bone marrow, and compensated for the hyperplasia of megakaryocytes in the model group. These results are consistent with the literature [28–30]. The number of platelet counts in peripheral blood increased in all icaritin-treated groups, bone marrow TPO expression decreased, and bone marrow megakaryocyte production decreased. Patients with acute myelogenous leukemia (AML) with high MPL expression exhibit low remission rates and poor prognosis and are prone to other hematological diseases, which suggests that MPL and TPO are associated with malignant hematopoietic cell growth [31]. The results of the present study demonstrated that MPL expression increased in the bone marrow of the model group compared to the control group and MPL decreased after drug administration. However, there was no significant difference in MPL expression in the spleen (results were not shown), which indicates that icaritin regulated bone marrow TPO generation, promoted bone marrow MPL expression, and inhibited the growth of malignant hematopoietic cells, and it also had no significant effect on the spleen TPO/MPL-related pathway.

The binding of TPO to receptors stimulates JAK2 (Janus kinase 2) phosphorylation, which phosphorylates the tyrosine residues at the distal end of the receptor and activates intracellular signaling pathways. Stat3 (signal transducer and activator of transcription) phosphorylates, forms dimers, translocates to the nucleus, regulates gene transcription, and regulates megakaryocyte and platelet production and apoptosis [9]. The results of bone marrow immunohistochemistry showed that the expression of p-Stat3 protein was increased in bone marrow megakaryocytes after modeling and the expression of p-Stat3 protein was decreased after administration, which indicates that platelets decreased after modeling, the JAK2/Stat3 pathway was activated, and megakaryocytes multiplied. JAK2/Stat3 pathway activation decreased after icaritin administration.

## 5. Conclusions

In summary, our study confirmed that icaritin increased PLT, PCT, and serum TPO levels, decreased the abnormal proliferation of megakaryocytes in the bone marrow and spleen, promoted platelet megakaryocyte formation, and improved megakaryocyte maturation in ITP. Our study confirmed that icaritin significantly ameliorated thrombocytopenia, promoted megakaryocyte proliferation, and facilitated the recovery of platelets and megakaryocytes of the hematopoietic system. TPO, MPL, and p-Stat3 levels in the bone marrow were measured for the first time in the ITP mouse model. This study shows that icaritin increased peripheral blood platelet counts in ITP mice and exhibited advantages, such as ease of action, low dose, low cost, and convenient administration, which make icaritin a promising drug candidate.

## Figures and Tables

**Table 1:** Blood routine of all groups.

Group	Control	Model	rhTPO	Y003-L	Y003-M	Y003-H
PLT	465.0 ± 23.2	293.9 ± 61.7^###^	645.1 ± 106.7^∗∗∗^	555.2 ± 45.1^∗∗∗^	536.8 ± 49.1^∗∗∗^	568.8 ± 94.3^∗∗∗^
PCT	0.166 ± 0.009	0.126 ± 0.020^###^	0.191 ± 0.064^∗^	0.226 ± 0.022^∗∗∗^	0.231 ± 0.021^∗∗∗^	0.254 ± 0.047^∗∗∗^
PDW	13.85 ± 0.40	13.78 ± 1.05	14.15 ± 0.54	13.88 ± 0.47	13.84 ± 0.15	14.11 ± 0.55
MPV	3.73 ± 0.07	4.56 ± 0.34^###^	4.30 ± 0.25	4.19 ± 0.15^∗^	4.38 ± 0.16	4.56 ± 0.17
WBC	5.79 ± 1.95	7.5 ± 1.19	8.16 ± 1.61	8.61 ± 2.41	7.25 ± 1.36	6.18 ± 1.93
RBC	8.98 ± 0.52	8.74 ± 0.38	8.57 ± 0.53	8.84 ± 0.27	8.52 ± 0.61	8.65 ± 0.53
HGB	141.0 ± 9.62	137.9 ± 5.77	133.3 ± 7.30	138.2 ± 4.23	135.0 ± 10.24	134.5 ± 8.18
HCT	39.59 ± 2.22	40.23 ± 1.91	39.26 ± 2.20	40.06 ± 1.44	38.75 ± 2.88	39.25 ± 2.45
MCV	44.11 ± 0.50	46.00 ± 0.81^###^	45.88 ± 0.67	45.34 ± 1.03	45.48 ± 0.55	45.36 ± 0.44
MCH	15.70 ± 0.32	15.77 ± 0.31	15.56 ± 0.21	15.67 ± 0.45	15.85 ± 0.24	15.53 ± 0.23
MCHC	356.1 ± 7.0	342.8 ± 7.4^##^	339.5 ± 6.0	345.4 ± 13.3	348.4 ± 8.8	342.6 ± 5.8
RDW	12.94 ± 0.35	14.32 ± 1.90	13.45 ± 0.41	13.68 ± 0.25	13.51 ± 0.30	13.52 ± 0.21
LYM	3.24 ± 1.81	3.54 ± 0.56	3.50 ± 1.24	4.40 ± 1.54	3.93 ± 0.84	2.75 ± 1.50
MID	0.66 ± 0.31	1.89 ± 0.68^##^	2.27 ± 0.49	2.39 ± 1.38	1.91 ± 0.36	2.39 ± 0.55

PLT: platelets; PCT: plateletcrit; PDW: platelet distribution width; MPV: mean platelet volume; WBC: white blood cell; RBC: red blood cell; HGB: hemoglobin; HCT: hematocrit; MCV: mean corpuscular volume; MCH: mean corpuscular hemoglobin; MCHC: mean corpuscular hemoglobin concentration; RDW: red cell distribution width; LYM: lymphocyte; MID: intermediate cell; GRN: granulocytes. The data are presented as the mean ± SD (*n* = 8 per group). ^##^*P* < 0.01 and ^###^*P* < 0.001 versus control group; ^∗^*P* < 0.05 and ^∗∗∗^*P* < 0.001 versus model group.

## Data Availability

The data used to support the findings of this study are available from the corresponding author upon request.
